# EPO for stroke therapy - Is there a future for further clinical development?

**DOI:** 10.1186/2040-7378-2-10

**Published:** 2010-05-12

**Authors:** Jens Minnerup, Heike Wersching, Wolf-Rüdiger Schäbitz

**Affiliations:** 1 Department of Neurology, University Hospital Münster, Albert-Schweitzer-Straße 33, 48149 Münster, Germany; 2 Institute of Epidemiology and Social Medicine, University of Münster, Domagkstraße 3, 48149 Münster, Germany; 3 Department of Neurology, Evangelisches Krankenhaus Bielefeld, Burgsteig 13, 33617 Bielefeld, Germany

## Editorial

The recently published double-blind, placebo-controlled, randomized phase II/III German Multicenter EPO Stroke Trial was conducted to evaluate the efficacy and safety of Erythropoietin (EPO) in stroke patients [1]. Of the 522 patients enrolled in this trial 460 were treated as planned (per-protocol population) with either EPO or placebo within 6 hours of symptom onset. The primary endpoint, change in Barthel Index on day 90, and all secondary outcomes failed to show any benefit of EPO. Moreover, an increased rate of intracerebral haemorrhages was observed after EPO treatment, resulting in an increased mortality in the EPO group. This effect was pronounced in patients who received EPO in addition to rt-PA.

In this Editorial we discuss potential reasons for the negative results of the German Multicenter EPO Stroke Trial, which contrasted the findings of a clinical pilot trial and several preclinical studies that showed beneficial effects of EPO [2,3]. Altogether we want to reflect on four major issues: 1. The overestimated efficacy of EPO in preclinical studies due to neglected quality characteristics in animal experiments. 2. An underpowering caused by the study design of the German Multicenter EPO Stroke Trial. 3. Unexpected side effects of EPO. 4. The future for a further development of EPO as a stroke drug.

So far EPO and EPO analogues were widely tested in animal stroke models [3]. In a meta-analysis of preclinical studies we analyzed the overall efficacy in focal cerebral ischemia. EPO and EPO analogues reduced infarct volumes by 32% and improved neurobehavioral deficits by 37% to 38%. However, Philip et al. recently showed that the quality of preclinical EPO studies as measured by a STAIR derived quality score was relatively low [4]. This is a crucial point because disregarding basic quality standards may cause an overestimation of a drug's efficacy in animal studies [5,6]. Indeed, this might be the case in experimental EPO studies. When animals were randomized to EPO treatment or placebo the efficacy was lower compared to studies in which randomization was not reported [7]. The way in which the outcome was assessed was identified as a further potential source of bias. When comparing studies that blindly assessed neurobehavioral deficits to studies with an unblinded assessment of outcome the latter reported a significantly higher efficacy of EPO [7].

The study design of the German Multicenter Stroke Trial is another potential reason for the failure to replicate the positive findings of prior preclinical and clinical studies. A particularly critical point is the allowed combination of rt-PA and EPO. This combination of treatments was neither investigated in animal models nor in the clinical pilot trial. Therefore adverse interactions of these two drugs as suggested by the increased rate of intracerebral haemorrhages in the German Multicenter Stroke Trial were unpredictable. A preceding investigation of EPO-rt-PA interactions could have had prevented a combination therapy in the clinical trial. In fact, a present mouse stroke study by Zechariah et al. showed that a combination of EPO and rt-PA induces blood-brain barrier permeability and extracellular matrix disaggregation [8]. However, it is uncertain whether the results of a single animal study based on surrogate markers would have influenced further clinical development of EPO, particularly in combination with rt-PA. When considering that an increasing number of stroke patients is treated with rt-PA within a therapeutical time window also adequate for neuroprotective therapies, the importance of thoroughly testing combination therapies in animal studies becomes evident [9]. This particularly includes investigations of whether the drugs interfere regarding their efficacy and safety. In addition, the allowed combination of EPO and rt-PA is critical for the issue of study power. Altogether, it is rather difficult to measure a beneficial effect on top of a highly effective therapy such as thrombolysis. The inclusion criteria also might have reduced the power: Patients with pre-existing disability were included in the study making it difficult to measure treatment-related differences on the primary outcome Barthel Index or on secondary outcomes such as the modified Rankin Scale. However, the pre-stroke Barthel Index and the pre-stroke modified Rankin Scale were not reported in the manuscript.

The negative findings of the German Multicenter EPO Stroke Trial could be a result of previously unknown side effects of EPO. So far, it was known that EPO increases the risk for myocardial infarction and composite end-points of death and cardiovascular events in patients with anaemia due to chronic kidney disease [10]. In addition, some years ago EPO was shown to enhance tumor progression and shorten survival in patients with some types of cancer [11]. Results of the more recent TREAT study suggest an intrinsic stroke-inducing capacity of EPO. In this study patients with diabetes, chronic kidney disease, and anaemia were randomly assigned to receive darbepoetin alfa or placebo [12]. Surprisingly, a significant higher number of strokes occurred in the darbepoetin alfa treated group compared to the placebo group. Unfortunately, it was not reported whether those strokes were ischemic or hemorrhagic [13].

The question arises what the disappointing results of the recent EPO trial mean for a future clinical development of the drug. One might consider that a further clinical stroke trial which excludes patients treated with rt-PA might show beneficial effects of EPO. Results of the German Multicenter EPO Stroke Trial, however, do not strongly support this assumption. In a subgroup analysis of non-rt-PA group none of the primary endpoints differed significantly between EPO and placebo treated patients. Only one secondary outcome measure, the delta NIHSS (NIHSS Day 1 minus Day 90) [1], revealed a better outcome after EPO treatment. In non-rt-PA treated patients there was even a tendency toward a higher death rate in the EPO group. The authors point out that this might be explained by the higher stroke severity of the dead patients on inclusion. Overall, the potential side effects of EPO will presumably prevent the conduction of further clinical stroke trials. However, non-haematopoietic EPO analogues remain as a therapeutic option for stroke, since the adverse effects of EPO were assumed to be mainly caused by its erythropoiesis stimulating effects. In a meta-analysis of preclinical studies we showed that non-hematopoietic EPO analogues are at least as effective as hematopoietic EPO-analogues [3]. The reason therefore might be EPO's mode of action in ischemic stroke, which is assumed to be based on a direct effect on neurons rather than on an increased hematopoiesis (for review see [14]). It was shown, that EPO receptors are expressed in the brain and that the neuronal EPO receptors which are distinct from those expressed by erythropoid precursors are stimulated by non-hematopoietic EPO analogues. Evidence regarding the safety of non-hematopoietic EPO analogues in stroke patients is expected in the near future since one clinical pilot trial of Carbamylated EPO in stroke patients was recently completed and another pilot trial has already started (http://www.clinicaltrials.gov/; NCT00756249 and NCT00870844). The future of non-hematopoietic EPO analogues for a further clinical development for stroke therapy will depend on the safety results of these trials.

## Competing interests

The authors declare that they have no competing interests.

## Authors' contributions

JM wrote the manuscript

HW wrote the manuscript

WRS revised the manuscript
